# The Influence of Extrusion Processing on the Gelation Properties of Apple Pomace Dispersions: Involved Cell Wall Components and Their Gelation Kinetics

**DOI:** 10.3390/foods9111536

**Published:** 2020-10-25

**Authors:** Vera Schmid, Heike P. Karbstein, M. Azad Emin

**Affiliations:** Institute of Process Engineering in Life Sciences, Food Process Engineering, Karlsruhe Institute of Technology (KIT), 76131 Karlsruhe, Germany; vera.schmid@kit.edu (V.S.); heike.karbstein@kit.edu (H.P.K.)

**Keywords:** plant cell-wall, by-product, rheological properties, complex viscosity, pectin, dietary fibers

## Abstract

By-products of fruits and vegetables like apple pomace can serve as techno-functional ingredients in foods. Due to their physicochemical properties, e.g., viscosity, water absorption, or oil-binding, food by-products can modify the texture and sensory perception of products like yogurts and baked goods. It is known that, by extrusion processing, the properties of by-products can be altered. For example, by thermo-mechanical treatment, the capacity of food by-products to increase viscosity is improved. However, the mechanism and involved components leading to the viscosity increase are unknown. Therefore, the complex viscosity of apple pomace dispersions and the involved fractions as pectin (a major part of the water-soluble fraction), water-soluble and water-insoluble fraction, were measured. In the investigated range, an increase in the pectin yield and water solubility was observed with increasing thermo-mechanical treatment by extrusion processing. However, pectin and water-soluble cell wall components had only a limited effect on the complex viscosity of apple pomace dispersions. The insoluble fraction (particles) were investigated regarding their swelling behavior and influence on the complex viscosity. An intensification of thermo-mechanical treatment resulted in increasing swelling behavior.

## 1. Introduction

Every year, between 3 and 5 million tons of apple pomace are estimated to be obtained from juice production worldwide [1,2]. Only a small amount of the by-product (~25%) is utilized as animal feed, for the recovery of pectin, and the extraction of bioactive compounds [3]. However, most of the apple pomace is treated as waste and disposed of in landfills, even though it is a very valuable resource of plant cell wall polymers [4,5].

Plant cell wall polymers of apple pomace are cellulose (~44%), hemicellulose (24%), pectin (12%), and lignin (20%) [4,6]. The proportions of the compounds can vary with the variety of apple and its ripeness. Cellulose, lignin, and some hemicelluloses, as well as some pectin, are usually defined as insoluble fibers (~36.5%), whereas the remaining pectin and hemicelluloses are defined as soluble fiber (~14.6%) [7,8,9].

Due to their nature, most food by-products have beneficial physicochemical properties, also known as techno-functional properties [5,10,11]. Among others, they can increase the water or oil holding capacity, work as a thickener or gelling agent, and thus modify the texture and sensory perception of food products [10,12]. Therefore, by-products like apple pomace have been used by researchers in various food products such as yogurt, cakes, snacks, bread, and meat products to improve the quality of these food matrices [6,9,13,14,15]. The application of food by-products meets consumers’ awareness and demand for more ingredients from natural resources, as well as for sustainable, health-promoting food with low environmental impact [10,12]. Thus, the use of apple pomace as a techno-functional ingredient is a promising way to utilize the large amount of apple pomace generated every year.

However, in its raw form, apple pomace only shows limited functionality. As they cause an undesirable texture and sensory perception of foods, their application is limited. Among others, extrusion processing is one option to modify the techno-functional properties of apple pomace. Previous studies have shown that its water solubility, thickening, and gelling properties can significantly be improved [16,17,18,19]. In particular, a change in the viscosity of dispersions of water and extruded by-products of citrus and apple is described [20,21]. However, the reason for the change in viscosity is still under investigation. It is assumed that the increased availability of pectin (a part of the soluble fraction) after extrusion processing is responsible for the increase in viscosity [22,23,24]. In this context, Ralet et al. [22] investigated the water-based extractability of pectin from lemon fibers after extrusion and their ability to form gels in the presence of sucrose and acidic pH. The extruded lemon pectin formed softer gels than commercial pectin [22], which is explained by a degradation in the backbone of pectin by extrusion processing [23]. The impact of other soluble cell wall polymers is not reviewed in the literature. By contrast, some studies dealing with orange pomace reported that the increase in viscosity is due to improved swelling properties of the insoluble fiber fraction [16,25].

Thus, there is a lack of knowledge regarding the mechanism for viscosity increase, and the relevant components, as well as their contribution. In this study, we investigated the gelation kinetics of water-soluble pectin, as well as the thickening behavior of the overall soluble and insoluble fractions of apple pomace. With a deeper understanding, the structure and, thus, the techno-functional properties of by-products can be modified by extrusion processing in a targeted nature.

## 2. Materials and Methods

### 2.1. Materials, Chemicals, and Reagents

Apple pomace was kindly supplied by Herbafood Ingredients GmbH (Company of H&F-Group, Werder (Havel), Germany). The residual moisture content of raw material was determined by Karl-Fischer titration using an automatic titrator (Titroline alfa, Schott Instruments GmbH, Mainz, Germany) to 2.5 ± 0.3% (*w*/*w*).

According to the supplier, the dietary fiber content was 60% and protein 5% (*w*/*w*). Apple pomace contained 11.6 g starch/100 g dry matter (DM). The total dietary fiber content was 67.6 ± 1.7 g/100 g dry weight, dividing into the insoluble dietary fiber content of 52.8 ± 1.2 and the soluble dietary fiber content of 12.5 ± 1.2 g/100 g dry weight. The analysis of monosaccharide composition showed that glucose (43.4 mol%) was the main monomer of insoluble dietary fibers, followed by arabinose (17.3 mol%). The main monosaccharide of soluble dietary fibers was arabinose (42.0 mol%), followed by galacturonic acid (32.9 mol%). More detailed information about chemical structures and composition are described in Schmid et al. [21]. The used chemicals (ethanol (96%), calcium chloride, sucrose, and citric acid) were of analytical purity grade and purchased from Carl Roth (Karlsruhe, Germany).

### 2.2. Extrusion Trials

All extrusion trials were carried out using a co-rotating twin extruder ZSK 26 Mc (Coperion, Stuttgart, Germany) with the same screw configuration containing reverse and kneading elements (detailed information about screw configuration see [26]). To apply various thermomechanical treatments, the screw speed and water content were varied. The process parameters were a barrel temperature of 100 °C (T_barrel_), screw speeds of 200, 450, and 700 min^−1^, and water contents of 42 and 17% at a total feed rate of 10 kg·h^−1^. The process conditions resulting from the various process parameters were characterized by material temperature T_M_ (before the die entrance) and specific mechanical energy input (SME). The SME was calculated according to the following Equation (1):

(1) $$SME\;\left(Wh\cdot kg^{-1}\right)=\frac{\frac{n}{n_{max}}\times \frac{M_{d}-\;M_{d,\;unload}}{100}}{\dot{m}}\;\times P_{max}$$

where *n* and *n_max_* are the actual and maximum screw speeds (1800 min^−1^), and *M_d_* and *M_d,unload_* are the actual and idle torques (%), respectively. $\dot{m}$ represents the total mass flow (1 kg·h^−1^) and *P_max_* the maximum engine power (40 kW).

### 2.3. Preparation of Dispersions

For rheological measurements, extruded samples were ground, sieved to 140–280 µm, and dried in a vacuum dryer (Heraeus, Hanau, Germany) to a constant mass at 25 °C. The dispersions were prepared in a beaker by adding 1 g apple pomace to 10 g demineralized water. The samples were sealed with paraffin film and stirred for 10 min at 25 °C on a Corning 6798-420D (Corning, New York, NY, USA) with 200 min^−1^. Afterwards, the samples were stored for 50 min before the rheological measurement started. 

### 2.4. Separation of Soluble Fraction

To gain the soluble fraction, the dispersions (preparation described above in Section 2.3) were centrifuged at 4250× *g* for 50 min at 25 °C (Centrifuge 5920R, Eppendorf, Hamburg, Germany). The supernatants were taken for rheological measurement (see Section 2.6).

### 2.5. Extraction of Pectin

For pectin extraction, 1 g of pomace and 39 g of demineralized water were mixed for 1 h and centrifuged at 4250× *g* for 50 min at 25 °C. The supernatant was removed by vacuum filtration (filter 11 µm pore size) and filled in a beaker. The pectin was extracted by adding 4 °C cold ethanol to the supernatant (ratio 3:1 (*w*/*w*)). The precipitated pectin was stored for 1 h before the precipitant was removed by vacuum filtration (filter of 22–25 µm pore size). The pectin was washed with 100 g ethanol and dried for at least 15 h. The extracted pectin was weighed. The extraction was performed at least 4 times. Afterwards, the pectin was crushed by a mortar and dissolved in demineralized water. The amount of water was selected according to the amount of water retarded after centrifugation. 

For the modified pectin solutions, 60% of D(+)-sucrose was added and the pH adjusted to ~pH 3 by citric acid. Alternatively, the pectin solutions were enriched with 0.15% calcium chloride.

### 2.6. Rheological Measurements

A Rheometer MC 301 (Anton Paar GmbH, Graz, Austria) was used to characterize the rheological properties and gelation kinetics. To avoid the destruction of the network, an oscillatory measurement routine was used. Therefore, the linear viscoelastic region was determined by amplitude sweeps at 1 Hz and was <1% first. The complex viscosity measurements of dispersions were performed by using a parallel plate system with a diameter of 50 mm. After adding the samples to the rheometer, the samples were put to rest for 90 s. The samples were measured in oscillatory mode with an amplitude of 0.1% and a frequency of 1 Hz at 25 °C. Afterwards, they were heated up to 80 °C in the rheometer with a heating rate of 5.5 K·min^−1^, followed by a cooling step to 25 °C with a cooling rate of 5.5 K·min^−1^. The complex viscosity was measured during this. 

The complex viscosity of the soluble fractions and pectin solutions was measured in a concentric cylinder system with a diameter of 27 mm due to the lower viscosity. All samples were measured in oscillatory mode with an amplitude of 0.1% and a frequency of 1 Hz at 25 °C.

The complex viscosity η* was calculated by the following Equation (2):

(2) $$\eta^{*}=\frac{\sqrt{{\left(G^{’}\right)}^{2}+{\left(G’’\right)}^{2}}}{\omega }$$

where ω is the frequency of oscillation (rad·s^−1^), *G′* the storage modulus (Pa), and *G″* the loss modulus (Pa). 

All measurements were performed in triplicate.

### 2.7. Preparation of Cross-Mixed Fractions

To gain more information about the role of the soluble and insoluble fractions and their interactions, aqueous dispersions based on (i) only the insoluble fraction of the extruded sample, and the (ii) insoluble fraction of the raw sample + soluble fraction of the extruded sample were prepared. The first step was to prepare the dispersions. Afterwards, they were centrifuged and separated as described above. For (i), the precipitant of the extruded sample was mixed with demineralized water. To keep the concentration constant, the amount of water added was equal to the amount of removed supernatant. Similarly for (ii), the supernatant of the extruded sample was added to the precipitate of the raw sample. The amount of the extruded supernatant added was equal to the amount of removed supernatant. Afterwards, the samples were sealed with paraffin film, stirred for 10 min at 25 °C and 200 min^−1^, and put to rest for 50 min before measuring.

### 2.8. Microscopy

A light microscope (Eclipse LV100ND, Nikon, Tokyo, Japan with DS-Fi1c camera) was used to analyze the swelling behavior of the insoluble fraction. The extruded apple pomace was dissolved in demineralized water and observed for almost 2 h at room temperature at 10- and 20-fold magnification. The experiments were performed twofold. The size of the particles was evaluated by using Image J (Version 1.52p). The particles were circled with the freehand selection before the area was measured.

### 2.9. Statistics

Data are presented as mean ± standard deviation (SD). ANOVA followed by the Holm–Sidak test was performed to determine statistical significance (*p* < 0.05) between groups using OriginPro2020 (OriginLab Corporation, Northampton, MA, USA).

## 3. Results and Discussion

### 3.1. Extrusion Conditions and Resulting Complex Viscosity

In our previous study [21], we showed that the complex viscosity of apple pomace can be increased by extrusion processing. Table 1 gives an overview of the process conditions and the resulting functional properties. By increasing the screw speed and reduction of water content, the thermo-mechanical treatment (represented by the specific mechanical energy and the material temperature) was increased. A low thermo-mechanical treatment was applied by high water contents of 42% at extrusion processing. The low thermo-mechanical treatment resulted in a decrease in viscosity of aqueous dispersions, which is presented as complex viscosity, whereas a high thermo-mechanical treatment (17% water content at extrusion and screw speed of 700 min^−1^) led to a complex viscosity of aqueous dispersion up to 48 times higher than raw material. In this study, these samples were used as a model system to perform an in-depth analysis of the role of the apple pomace components on the significant changes in the rheological behavior of their aqueous dispersions.

To characterize the crucial components for the formation of high viscous dispersions, the impact of each fraction on the viscosity was investigated. First, the effect of pectin, which is part of the soluble fraction, on the viscosity was investigated. Secondly, the effect of the overall soluble fraction, containing pectin, hemicellulose, and small amounts of mono-/disaccharides and protein, on the viscosity was studied. Thirdly, the swelling behavior of insoluble particles was examined. Last, the interplay of both soluble and insoluble fractions was discussed. 

### 3.2. Influence of Pectin on the Complex Viscosity of the Continuous Phase

#### 3.2.1. Yield of Pectin after Extrusion Processing

**Figure 1:**
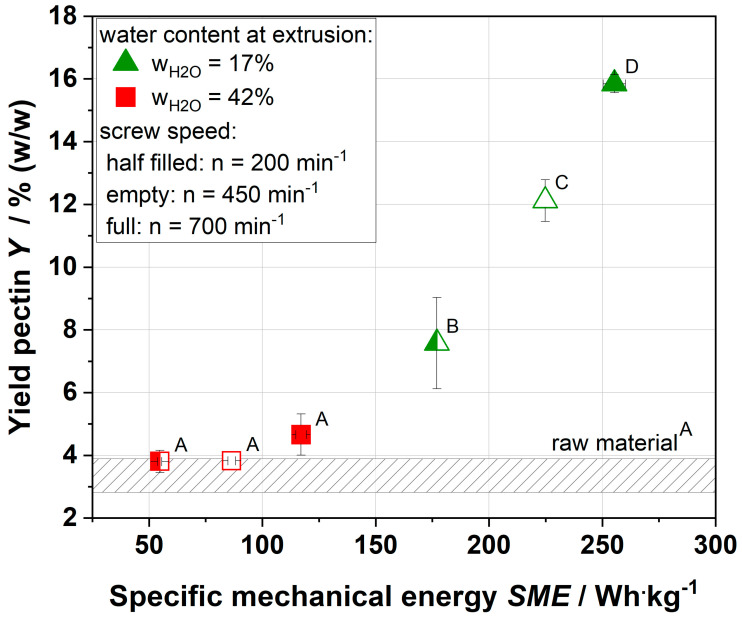
Dependency of specific mechanical energy (SME) and yield of water-soluble pectin extracted by ethanol precipitation (pectin yield) in weight percentage for raw apple pomace and extruded pomace. The apple pomace was extruded at water contents of 17 (triangular) and 42% (square), and 200 (half-filled), 450 (empty), and 700 (full) min^−1^. Different symbols were chosen to uniquely identify samples and their process conditions in the following figures. ^A–D^ Mean values within a row that are marked with different letters differ significantly (*p* < 0.05).

The water-extractability of pectin from raw and extruded apple pomace was studied at 25 °C. As the extruded samples with 42% water content showed lower viscosity than raw material, the extruded samples with 17% water content showed significantly higher viscosity, and these samples were chosen for pectin extraction. Further, to identify the impact of screw speed on the yield of pectin, screw speeds of 200, 450, and 700 min^−1^ were investigated. The influence of specific mechanical energy on the pectin yield is shown in Figure 1. 

For the raw material, only 3–4% of pectin (*w*/*w*) was extracted. A similar extraction yield was measured for samples extruded at mild thermo-mechanical conditions (water content of 42%, screw speeds of 200 and 450 min^−1^). By increasing the specific mechanical energy by increasing the screw speed to 700 min^−1^, the extraction yield was slightly increased to ~5%. A further increase in the thermo-mechanical treatment by the reduction in water content (17%) resulted in a significant increase in the pectin yield. At a screw speed of 200 min^−1^, a pectin yield of ~8% was obtained, and at 700 min^−1^, a pectin yield of ~16% was obtained. Ralet et al. [22,23] also reported an increase in the yield of pectin after extrusion for lemon cell walls. By increasing the specific mechanical energy from 170 to 236 kWh·t^−1^, a doubling of pectin yield after extrusion processing was measured (12.5–29.4%), which is in line with the values of this study. 

The dependency between increasing thermomechanical treatment and the extractability of cell wall pectin indicated that extrusion processing had modified the cell wall structure. Probably, the middle lamella of the cell wall, which contains a high amount of pectin, was susceptible to thermomechanical treatment. As more pectin can be extracted after extrusion processing, this suggested that pectin plays a major role in the formation of high-viscosity gels.

To investigate the contribution of pectin and its increase in the complex viscosity of the entire system, pectin was resolved in demineralized water and the gelation kinetics determined. 

#### 3.2.2. Effect of Pectin on the Complex Viscosity of the Continuous Phase

**Figure 2:**
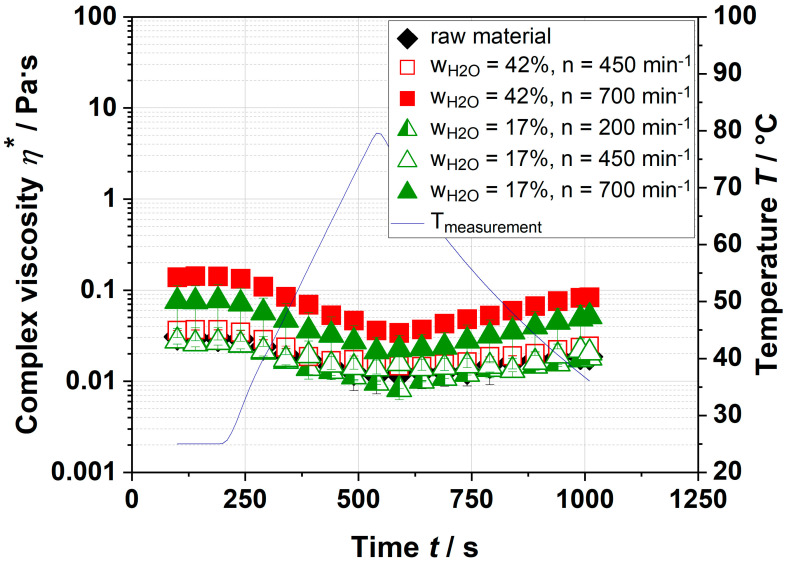
Gelation kinetics of extracted and resolved pectin of raw material and extruded apple pomace for various process parameters. The blue line depicts the temperature ramp of measurements.

Due to some pectin needing a certain temperature to gel, the temperature was increased during the measurements. Figure 2 shows that the complex viscosity of the continuous phase of extracted and resolved pectin only changed slightly by a temperature increase and decrease. By increasing the temperature, the complex viscosity decreased slightly and rose again when the temperature dropped. However, no gelation (defined as η_end_ >> η_start_) was observed.

Thus, it can be assumed that pectin was not able to form a spanning network to increase the complex viscosity to a similar extent as the dispersion of apple pomace did (Table 1). The low complex viscosity could be related to a difference in pH, sugar, and ion content compared to the aqueous dispersion of the whole sample. It is well-known that highly methyl-esterified pectin needs sugar and acid; low methyl-esterified pectin needs CaCl_2_ to form networks or gels [27,28]. 

**Figure 3:**
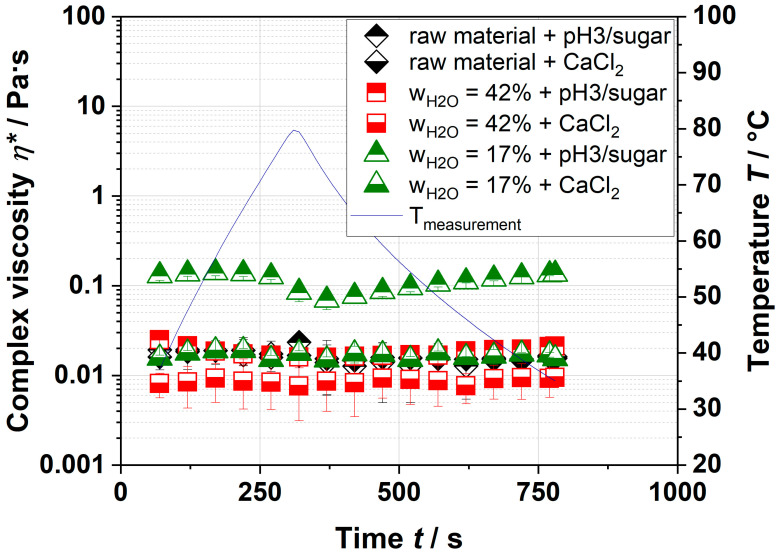
Gelation kinetics of extracted and resolved pectin with the addition of CaCl_2_ or sugar at pH 3. The extrusion process parameters of the extruded sample were water contents of 42 and 17%, and a screw speed of 700 min^−1^. The blue line depicts the temperature ramp of measurement.

To verify this approach, dispersions with various pH, sugar, and ion content were prepared and analyzed. In plant cell walls, pectin is present as a highly methyl-esterified polymer [29]. Therefore, it was assumed that the reduction in pH and the addition of sugar increased the complex viscosity. The results of pectin with additives are depicted in Figure 3. No significant differences in the complex viscosities, which vary between 0.008–0.2 Pa·s, were observed.

Neither with the addition of CaCl_2_ nor by the reduction in the pH value plus sugar addition was an increase in complex viscosity observed (Figure 3). However, the complex viscosities of various extruded apple pomace dispersions were ~300 Pa·s (see Table 1), which cannot be related to the increased yield of pectin. A possible explanation might be the absent nonpectinaceous soluble fraction (e.g., hemicelluloses, proteins) and/or the insoluble fraction, which can contribute to the viscosity of the aqueous solution. Their influence on the complex viscosity was investigated in the following sections.

### 3.3. Influence of Soluble Fraction on the Complex Viscosity of the Continuous Phase

**Figure 4:**
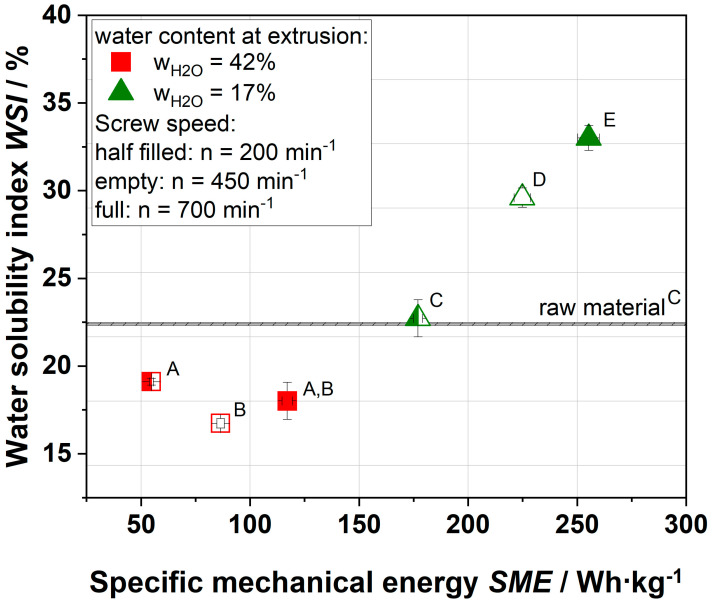
Effect of specific mechanical energy (SME) on the water solubility index (WSI) of apple pomace. The apple pomace was extruded at water contents of 17 (triangular) and 42% (square); screw speeds were 200 (half-filled), 450 (empty), and 700 (full) min^−1^. ^A–E^ Mean values within a row that are marked with different letters differ significantly (*p* < 0.05).

Figure 4 shows the water solubility index (WSI) of raw material in comparison to extruded samples (data published in [21]). The water-soluble part contains mainly pectin, but also hemicelluloses and several mono- and disaccharides. 

The WSI of raw apple pomace was 22.4%. At low thermo-mechanical treatment and high water content (42%), the water solubility decreased to lower values than the WSI of raw material. An increase in thermo-mechanical treatment resulted in increased WSI values as well. The decrease in WSI of samples extruded at 42% water was probably due to agglomeration of apple pomace while extruding. The increase in WSI with increasing thermo-mechanical treatment was due to the destruction of the cell wall. Mainly, pectin, hemicelluloses, and small fractions of cellulose, which were dissociated by extrusion processing, were solubilized. 

**Figure 5:**
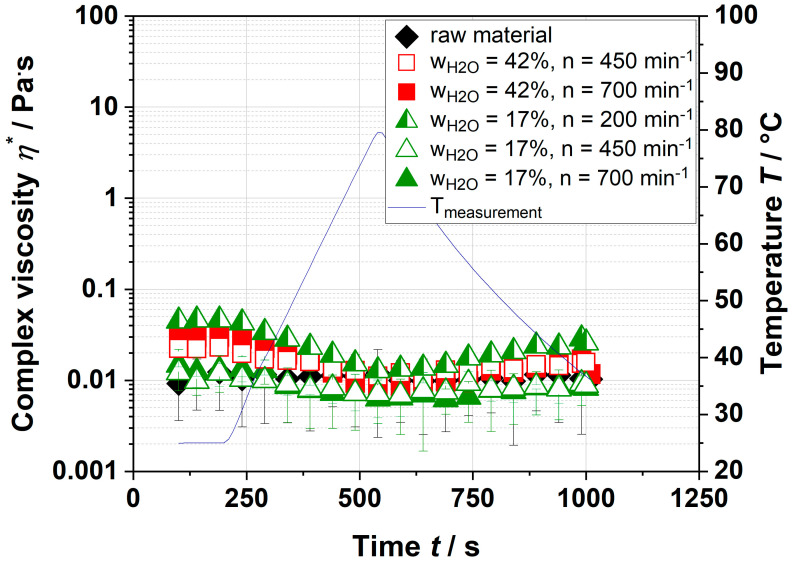
Rheological behavior of the continuous phase for raw material and various extruded samples. The extrusion process parameters of the extruded sample were water contents of 42 and 17%, and a screw speed of 700 min^−1^. The blue line depicts the temperature ramp of measurement.

The impact of the overall soluble fraction of the raw material and various extruded apple pomace samples on the complex viscosity of the continuous phase is shown in Figure 5. 

Trends and absolute values were comparable to those seen for extracted pectin (see Figure 2 and Figure 3), which is a part of the soluble fraction. The values of complex viscosity remained almost unchanged and had similar values as that of pure water. Even samples with a higher amount of soluble fraction (~33%, ∆) did not show a significant increase in the complex viscosity. Thus, it can be concluded that the water-soluble fraction of pectin, hemicellulose, and several mono- and disaccharides was not able to form a spanning network to increase the viscosity. 

### 3.4. Influence of Insoluble Fraction on the Complex Viscosity

**Figure 6:**
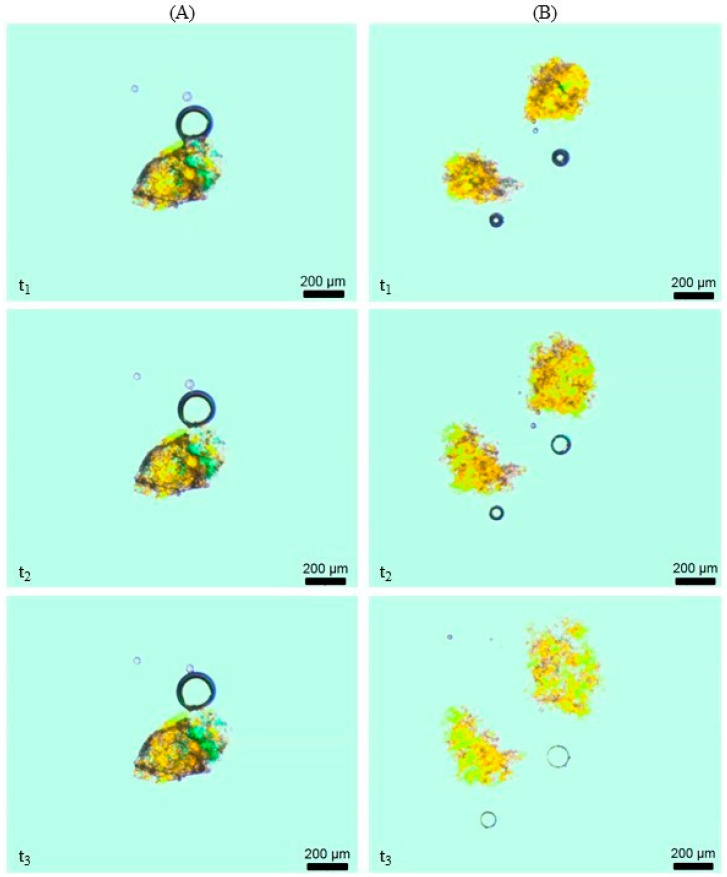
Swelling of particles: Raw material (**A**) and extruded sample (**B**) (W_H2O_ = 17%) at time t_1_ = 0 min, t_2_ = 30 s, and t_3_ = 30 min.

The insoluble particles mainly consist of cellulose, lignin, and some hemicelluloses. The growth of the insoluble fraction or representative particles in water is shown in Figure 6. 

**Figure 7:**
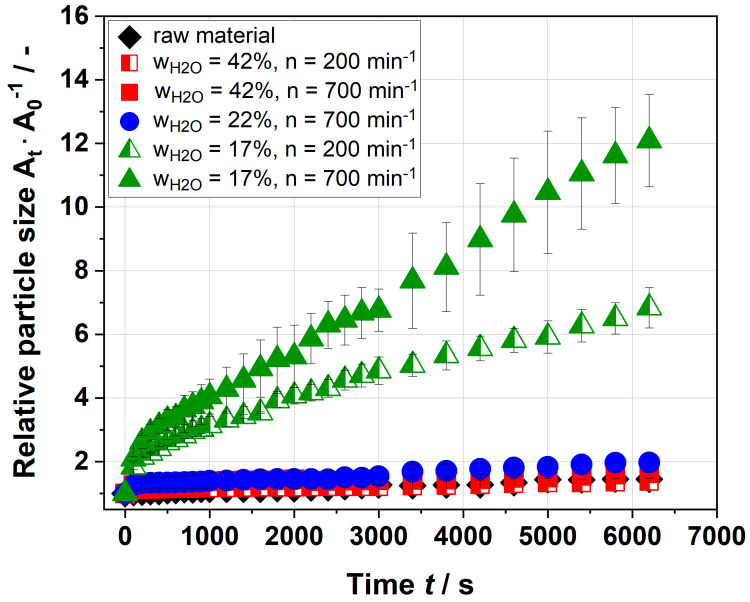
Growth of insoluble particles in water by microscopic image analysis. The extrusion process parameters of the extruded sample were water contents of 42 and 17%, and a screw speed of 700 min^−1^. The relative particle size A_t_ · A_0_^−1^ is the area a particle covers at time t divided by the initial area a particle covers at time t = 0 s.

The size and shape of the particles of raw material (A) did not change as time passes. By contrast, already 30 s after the start, differences in size and shape of extruded particles were observed. After 30 min, a further swelling of the particles was noticed. To compare the swelling behavior of various extruded samples, the change in particle size was visualized. The growth of particles is plotted in Figure 7. As the microscope takes two-dimensional pictures, the shown data represent a plane expansion only.

The particle size of the raw material changed only slightly from 1 to 1.45. Similar values were observed for samples extruded at a water content of 42%. As a consequence of extrusion processing at a water content of 22%, and a screw speed of 700 min^−1^, the size of these particles almost doubled after 2 h. The most prominent increase in size showed particles that were extruded at a water content of 17%. An applied screw speed of 200 min^−1^ resulted in particles that grew to almost 7 times the initial size (t = 6600 s). Particularly, a strong increase was observed in the first seconds. After 100 s, the particles already doubled in size. Applying a screw speed of 700 min^−1^, the particles grew by approx. 12 times. Due to a very strong swelling in the first seconds and the fact that the sample preparation (placing the sample under microscope and focusing) took some time, the initial value of this sample was probably underestimated. Therefore, the initial size of the sample was extrapolated.

A swelling of extruded orange pomace was already observed by Huang et al. and Larrea et al. [16,25]. However, the swelling capacity was much lower and no kinetics were determined. The strong swelling in the first seconds, observed in this study, could be explained by the increase in the macromolecular porosity of the particles by extrusion processing [21]. The increase in porosity may be explained by a disruption of the cell wall architecture. Extrusion processing probably disrupts the hemicellulose network and thus loosens the cell wall. The high porosity might accelerate the absorption of water. However, the presented results contradict the hypothesis of Redgwell et al., who assumed that a higher *SME* leads to smaller and denser particles, which absorb water sluggishly [20].

### 3.5. Dispersion with Solid and Liquid Fraction/Cross-Mixed Fraction

**Figure 8:**
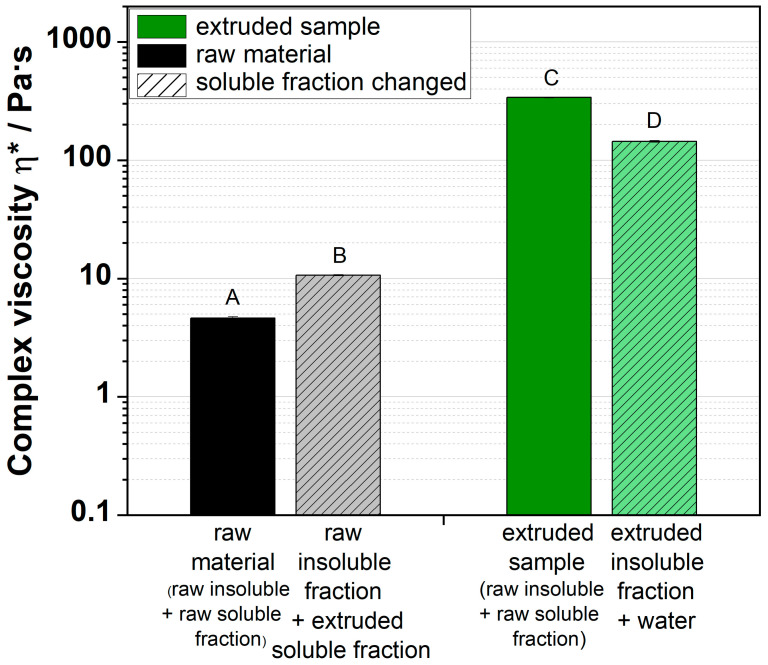
Change in complex viscosity of dispersions of raw material, extruded sample (W_H2O_ = 17%, *n* = 700 min^−1^) (full), and samples with a replaced soluble fraction (soluble fraction of treated sample/new water) (dashed). ^A–D^ Mean values within a row that are marked with different letters differ significantly (*p* < 0.05).

The presented results showed that swelling of the particles has a huge impact on the complex viscosity of extruded apple pomace dispersions (Figure 6 and Table 1). However, the observation of swelling is not evidence that the insoluble fraction is responsible for the viscosity increase. In microscopic image analyses, the effect of soluble fraction, which is still a part of the particle and which increased with thermomechanical treatment, was neglected. To identify whether the soluble or insoluble fraction or their interplay was responsible for the increase in complex viscosity, the procedure described in the following was chosen: The dispersion was centrifuged and the supernatant was removed. The precipitant of the extruded apple pomace was mixed with demineralized water (light green dashed). The raw material was also centrifuged and separated. Afterwards, the proportionate amount of supernatant of the dispersion showing a high viscosity was added (grey dashed). The complex viscosities of these dispersions were measured and compared to the complex viscosities of the initial dispersions (Table 1). The results are shown in Figure 8.

The complex viscosity of the raw material increased by adding the soluble fraction of an extruded sample (grey dashed) compared to the original raw material (black). The complex viscosity of the dispersions containing only the insoluble fraction of the extruded sample and new water (light green dashed) decreased compared to the initial complex viscosity of the extruded sample (green). This decrease depended on the processing conditions (results not shown). At mild thermo-mechanical treatment conditions, e.g., low screw speed, the decrease in the complex viscosity was not as significant as at more severe conditions. Small declines in the complex viscosity were observed for lower thermo-mechanical treatment or lower screw speed, respectively.

These observations stated that both the soluble and the insoluble fraction contribute to the formation of viscous dispersions. It seems that the insoluble fractions, which build the dispersed phase, were the main cause leading to high viscous dispersions. Probably, by extrusion processing, the cell wall architecture was altered and, by consequence, the macromolecular porosity of the particles increased. This leads to better swelling behavior of the particles. The swollen particles form a network, which is probably strengthened by the soluble molecules.

## 4. Conclusions

As raw and extruded apple pomace increase the viscosity of dispersions, from a technological point of view, it can be used as a techno-functional and thickening ingredient in foods. The results of this study give insights into the thickening behavior of extruded apple pomace and the causes. 

Although extrusion treatment resulted in an increased extractability of soluble components (pectin, overall water-soluble polysaccharides), the soluble fraction alone showed no significant change in the viscosity of aqueous dispersions. The present study shows that besides the solubilization of cell walls, extrusion processing increased the water absorption and swelling behavior of insoluble apple pomace particles. This was identified to be the main cause of significant increases in complex viscosity of aqueous pomace dispersions. Intensive thermo-mechanical treatment by extrusion processing resulted in particles that swelled up to ~12 times their initial size. Particularly, a tremendous increase in size was observed in the first minute. The cross-mixing of the soluble and insoluble fraction showed that the interplay of soluble and insoluble fraction determines the final complex viscosities of aqueous apple pomace dispersions.

## Figures and Tables

**Table 1:** The process parameters (specific mechanical energy (SME), material temperature (T_M_)) and complex viscosity of raw and extruded apple pomace from extrusion trails at a barrel temperature of 100 °C and a total feed rate of 10 kg·h^−1^ (published in [21]).

	Specific Mechanical Energy SME/Wh·kg^−1^	Material Temperature at Die Entrance T_M_/°C	Complex Viscosity η*/Pa·s
raw material	-	-	4.63 ± 0.13
water content 42%, 200 min^−1^	54.8 ± 0.9	112.2 ± 0.1	0.11 ± 0.00
water content 42%, 450 min^−1^	86.5 ± 1.7	128.0 ± 0.1	0.07 ± 0.00
water content 42%, 700 min^−1^	117.1 ± 2.3	137.3 ± 0.1	0.31 ± 0.00
water content 17%, 200 min^−1^	177.0 ± 2.1	140.2 ± 0.1	218.57 ± 1.68
water content 17%, 450 min^−1^	224.8 ± 3.7	156.7 ± 0.2	207.84 ± 2.19
water content 17%, 700 min^−1^	255.3 ± 4.9	164.0 ± 0.1	338.66 ± 3.56
